# Implementation of a Specific Set of Intraoperative C‐Arm Fluoroscopy Terminologies in Percutaneous Vertebroplasty

**DOI:** 10.1111/os.13824

**Published:** 2023-10-09

**Authors:** Yanchun Xie, Hongwen Gu, Yongcun Wei, Anwu Xua, Hailong Yu

**Affiliations:** ^1^ Department of Orthopedics General Hospital of Northern Theater Command Shenyang China; ^2^ Dalian Medical University Dalian China

**Keywords:** C‐arm, Fluoroscopy, Repeat Puncture Rate, Vertebroplastic, Vertebroplasty

## Abstract

**Objective:**

Percutaneous vertebroplasty (PVP) is currently the primary minimally invasive surgical approach for treating vertebral compression fractures caused by senile osteoporosis. The current existing problem is the lack of research on the application of a specific set of intraoperative C‐arm fluoroscopy terminologies in PVP. Therefore, the purpose of this study is to explore the use of a specific set of intraoperative C‐arm fluoroscopic terminologies in PVP in order to increase fluoroscopy accuracy, decrease fluoroscopy frequencies and ray protection, and minimize operation times through rapid preoperative training of surgeons and radiographers.

**Methods:**

Spine surgeons and radiographers with at least 5 years of experience from nine different hospitals were randomly selected for a series of specialized intraoperative C‐arm fluoroscopy terminology training between October 2018 and December 2021. Before and after the training, they were surveyed using a five‐point Likert scale to statistically compare their knowledge of the terminology. Simultaneously, 190 PVP cases completed by these surgeons and radiographers before and after the training were chosen for comparison and analysis of fluoroscopy times, effective fluoroscopy rate, fluoroscopy time, repeated puncture rate, and other indicators before and after receiving specialized terminology training. Two‐sample tests were mainly used to investigate differences in answers between surgeons and radiographers.

**Results:**

After the training, there was a notable improvement in the fluency of intraoperative communication between professional spine surgeons and radiographers. By comparing the indicators of pre‐training with post‐training, the effective anteroposterior fluoroscopy rate increased from 46.5% to 75.7%; the effective lateral fluoroscopy rate increased from 59.8% to 76.9%. Moreover, a notable decrease in communication barriers, fluoroscopy frequencies, fluoroscopy time, and the rate of repeated punctures, and a notable increase in the effective fluoroscopy rate was observed.

**Conclusion:**

Smooth intraoperative communication between professional spine surgeons and radiographers can significantly lower the communication barrier, reduce the fluoroscopy frequencies and time, the rate of repeated puncture, and increase the effective fluoroscopy rate, all of which are important in improving the fluoroscopy in PVP.

## Graphical abstract

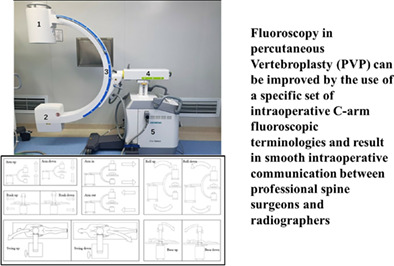

## Introduction

Osteoporotic fractures are widespread among the elderly, with spinal compression fractures being the most common form. Compression and rupture of the vertebral body after a fracture can cause local kyphosis deformity and throw off the sagittal balance. With its minor trauma, minimally intrusive nature, and speedy recovery time, PVP is currently the primary minimally invasive surgical approach for treating vertebral compression fractures caused by senile osteoporosis. In a survey of 212 orthopaedic surgeons and 235 radiographers, 97% of orthopaedic surgeons and 85% of radiographers used a C‐arm fluoroscopic machine in the operating room for daily work.^1^ However, 89% of orthopaedic surgeons and 91% of radiographers who responded to the questionnaire were without specific C‐arm terminology training.^2^ Through existing literature,^3^, ^4^, ^5^, ^6^ we found that intraoperative fluoroscopy terminologies could improve the communication efficiency between operators and radiographers, but there was no unified specific term. Moreover, the terms were not in accordance with the movement's definition of the control joint of the C‐arm.

Intraoperative C‐arm fluoroscopy is primarily employed for the completion of domiciliary PVP to minimize or lessen the aforementioned issues. There is currently no report on the professional terminologies and special envoy standards for the use and placement of the C‐arm by surgeons and intraoperative radiographers, which leads to a number of issues, including bone cement leakage, spinal cord nerve damage, and radiation exposure.^7^, ^8^, ^9^, ^10^, ^11^

The current standard C‐arm has the capability to move in 12 directions. A major cause of poor fluoroscopic accuracy is, first, shaky communication between surgeons and intraoperative radiographers. There is a disconnect between the surgeons' ability to articulate their needs and the radiographers' ability to interpret those needs. Second, if the fluoroscopy is not precise enough, the surgeon might misjudge the results and injure a blood vessel or a nerve while operating. This would be because the fluoroscopy would have to be repeated more often, or the target segment would not be in its standard anatomical position during the first try. Third, medical personnel and patients may be at risk for developing disorders associated with radiation exposure if they are repeatedly subjected to high fluoroscopy frequencies. Last but not least, because patients over the age of 65 with osteoporosis are also typically over the age of 65 with other medical conditions, a prolonged fluoroscopy session can raise perioperative risk and patient tension and anxiety. There is currently no report detailing the agreed‐upon definitions for C‐arm fluoroscopy used in PVP. This study aims to increase fluoroscopy accuracy, decrease fluoroscopy frequencies and ray protection, and minimize operation times through rapid preoperative training of surgeons and radiographers by proposing a set of intraoperative C‐arm fluoroscopy techniques.

## Methods

### Multi‐Centre Questionnaire Survey

From October 2018 to December 2021, 20 professional spine surgeons and 20 radiographers who have worked in nine hospitals for more than 5 years were selected for the five‐point Likert scale (to measure the attitudes or views of the subjects) to assess the perceived quality of pre‐training of a set of specific intraoperative C‐arm fluoroscopy terminology and postoperative communication. It consists of a statement or a question, followed by a series of five or seven answer statements. Respondents choose the option that best corresponds with how they feel about the statement or question.^12^ This study was approved by the hospital's institutional review board (IEC No. 56379) and was conducted per the ethical principles of Helsinki. Written informed consent was obtained from each participant.

All operations of C‐arm machines PLX7200 (produced by Siemens in Germany) used in the study were trained by a professional technician for 1 hour. The survey results were collected pre‐training and post‐training, and descriptive statistics were made for all questions in the survey.

### The Training

The study gathered data on 190 PVP procedures performed at nine different hospitals and compared fluoroscopy‐related indicators between surgeons and radiographers before and after training. According to the study's proposed notion of efficient fluoroscopy, radiographers' displays of fluoroscopy pictures must conform to the anteroposterior fluoroscopy standard. The target vertebral body has a centred spinous process, bilateral pedicles of equal length, and an upper or lower endplate parallel to the C‐projection arm's direction. The upper or lower endplate of the target vertebral body is parallel to the projection direction of the C‐arm, and the specific target vertebral body (wedge‐shaped vertebral body) or L5‐S1 segment is shown. The training was mainly focused on the following four aspects.

**FIGURE 1:**
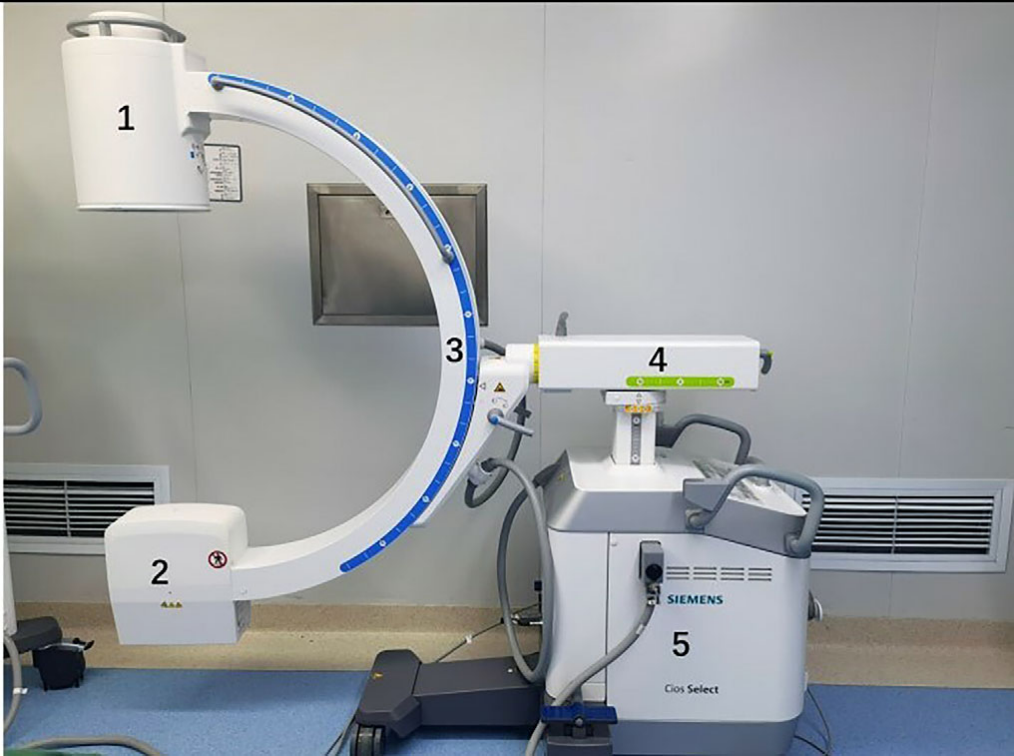
The six parts of the C‐arm joints. 1. Imaging system; 2. Tube; 3. Arm body; 4. Horizontal support frame; 5. Vehicle body; 6. Arm body is as front part, and the vehicle body is the rear part.

**FIGURE 2:**
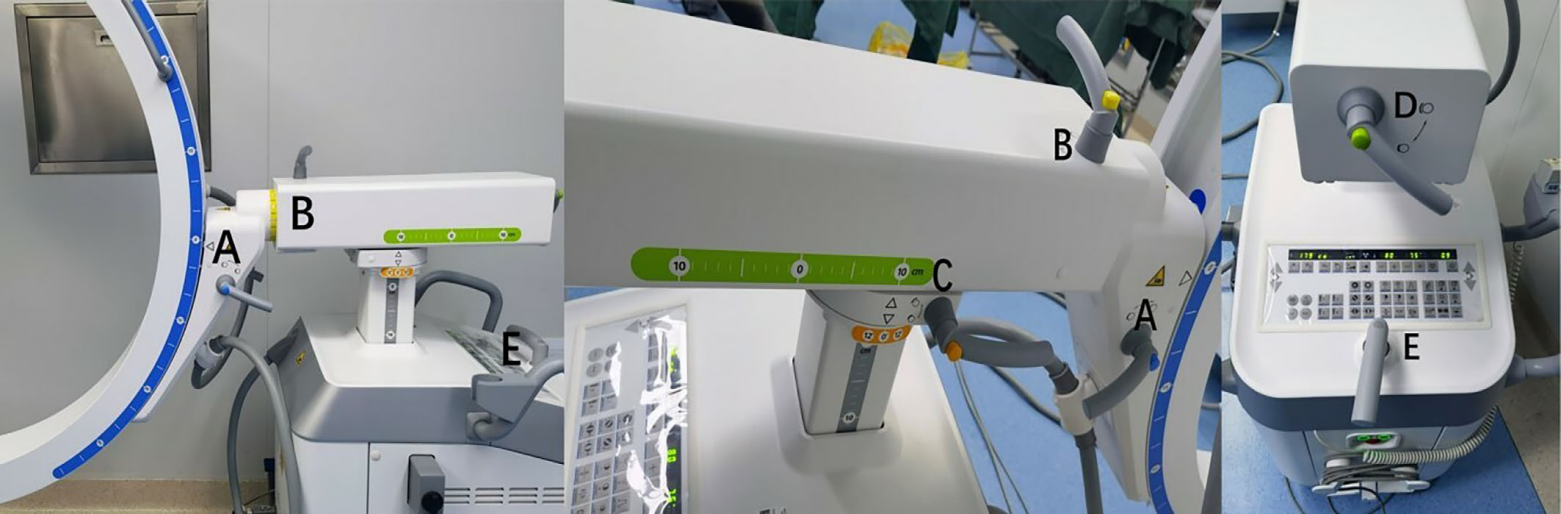
Five movement joints of the C‐arm. Joint A: To adjust the vertical‐horizontal rotation movement between the imaging system and the ball tube. Joint B: To adjust the rotation movement between head tilt and foot tilt of the arm body. Joint C: On the premise that the vehicle body position is fixed, adjust the horizontal support frame's left and right rotation movement relative to the vehicle body. Joint D: On the premise that the vehicle body position is fixed, adjust the horizontal support frame to travel forward and backwards relative to the vehicle body. Joint E: To adjust the movement of the vehicle body in all directions.

**FIGURE 3:**
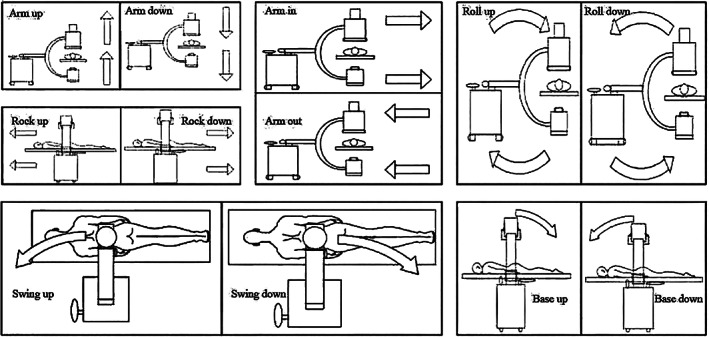
C‐arm movements in 12 relative directions. On the premise that the vehicle body position is fixed, raise the arm body vertically by pressing the “raise” button of the C‐arm; the decrease operation procedure is the reverse of the elevation operation procedure, and the raised distance and decreased distance are all measured in cm for operation.

1. The six parts of the C‐arm joints:
  - Imaging system.
  - Tube.
  - Arm body.
  - Horizontal support frame.
  - Vehicle body.
  - Arm body is as front part, and the vehicle body is the rear part (Figure 1).
2. Five movement joints of the C‐arm:
  Joint A: To adjust the vertical‐horizontal rotation movement between the imaging system and the ball tube.
  Joint B: To adjust the rotation movement between head tilt and foot tilt of the arm body.
  Joint C: On the premise that the vehicle body position is fixed, adjust the horizontal support frame's left and right rotation movement relative to the vehicle body.
  Joint D: On the premise that the vehicle body position is fixed, adjust the horizontal support frame to travel forward and backward relative to the vehicle body.
  Joint E: To adjust the movement of the vehicle body in all directions (Figure 2).
3. C‐arm movements in 12 relative directions:
  - Arm up and arm down (cm): On the premise that the vehicle body position is fixed, raise the arm body vertically by pressing the “raise” button of the C‐arm; the decrease operation procedure is the reverse of the elevation operation procedure, and the raised distance and decreased distance are all measured in cm for operation (Figure 3).
  - Arm in arm out (cm): The arm body moves forward in the direction away from the vehicle body; this movement is called “arm in” otherwise, it is called “arm out,” which is mainly by adjusting the joint E to move the vehicle body, or by adjusting the joint D to move the horizontal support frame relatively to move the vehicle body as supplementary means (Figure 3).
  - Rock up and rock down (cm): The movement to the head end of the patient is called “rock up,” and the movement to the foot end of the patient is called “rock down.” During the process of movement from rock up to rock down, the walking direction of the C‐arm shall be parallel to the patient's trunk, the moving distance is measured in centimeters, and the operation is performed by adjusting joint E (Figure 3).
  - Base up and base down (°): The movement is only used for anteroposterior fluoroscopy. By adjusting joint B, the tube on the ventral end of the trunk moves to the head of the patient, and the imaging system on the dorsal end of the trunk moves to the foot end of the patient, which is called “base up,” otherwise it is called “base down,” and the moving distance is measured in angle (Figure 3).
  - Roll up and roll down (°): The movement is only used for frontal fluoroscopy. By adjusting joint A, the tube on the ventral end of the patient's trunk slides towards the vehicle body, and the imaging system on the back end of the patient's trunk slides away from the vehicle body, which is called “roll up.” Otherwise, it is called “roll down,” and the moving distance is measured in angle.
  - Swing up and swing down (°): The movement is only used for lateral fluoroscopy. By adjusting the joint E and joint C and with the vehicle body as the centre of the dot, the tube at the far end of the vehicle body moves to the patient's head end, which is called “swing up,” otherwise it is called “swing down.” (Figure 3).
4. Anteroposterior fluoroscopy standard: The spinous process of the target vertebral body is centred, the bilateral pedicles are symmetrical and equal, and the upper or lower endplate of the vertebral body is parallel to the projection direction of the C‐arm. The upper or lower endplate of the target vertebral body is parallel to the projection direction of the C‐arm, and the specific target vertebral body (wedge‐shaped vertebral body) or L5‐S1 segment is shown.

### General Case Data

For this study, we gathered data from the aforementioned facilities between October 2018 and December 2021, collecting a total of 190 patients treated by expert spine surgeons and radiographers (90 before and 100 after the training). All 190 patients had single‐segment thoracolumbar fractures that were treated with local anesthetic by board‐certified spine surgeons and radiographers.

A total of 190 patients (79 male and 111 female) were enrolled in the study (16 patients were excluded according to the below exclusion criteria), with ages ranging from 61 to 94 (with 74.39 being mean).

The inclusion criteria were (1) previous PVP treatment; (2) completed imaging data (including X‐ray, MRI, CT); (3) VAS (visual analog scale) score higher than 6; and (4) patient request of operative treatment and refusal of conservative treatment.

The exclusion criteria were (1) patients with evidence of bone tumors and other bone metabolic diseases; (2) CT shows an incomplete or ruptured posterior wall of a fractured vertebral body; and (3) the presence of surgical contraindication.

This cohort was divided into four cases of the 7th thoracic vertebral fracture, five cases of the 8th thoracic vertebral fracture, nine cases of the 9th thoracic vertebral fracture, 21 cases of the 10th thoracic vertebral fracture, 31 cases of the 11th thoracic vertebral fracture, 52 cases of the 12th thoracic vertebral fracture, one lumbar vertebral fracture, 42 cases of the first lumbar vertebral fracture. The number of anteroposterior fluoroscopies, the number of effective durations in the anteroposterior position, the number of lateral fluoroscopies, and the effectiveness of lateral fluoroscopy were recorded, and the C‐arm fluoroscopy was broken down into positioning fluoroscopy, puncture fluoroscopy, tube drainage fluoroscopy, and fluoroscopy of bone cement injection. Indicators such as the total amount of time spent on anteroposterior and lateral fluoroscopy, the frequency with which punctures were repeated, the needle insertion success rate, and the needle insertion failure success rate were also studied.

### Statistical Analysis

Descriptive statistics of all the questions in the multi‐centre questionnaire survey were carried out in SPSS software (28.0.1.0, IBM, US). Two‐sample tests were used for baseline comparison to investigate differences in answers between surgeons and radiographers. In addition, a two‐sample t‐test was used to compare the answers of the entire survey cohort (surgeons and radiographers) and the pre‐training survey and post‐training survey of each group. A two‐sample t‐test was also conducted to compare the number of fluoroscopy frequencies, fluoroscopy time, and puncture rate pre‐training and post‐training. *p* values less than 0.05 were considered statistically significant.

## Results

### Intraoperative Communication Level

Intraoperative communication levels increased after a term of training. There were 20 participants from the 5‐point Likert scale survey, including experienced spinal surgeons and radiological technologists. Intraoperative communication with the radiographer was rated as significantly improved (0.398), and the trainees reported significant reductions in the difficulty of communicating C‐arm movement terms, the need for motor correction, and the difficulty of communicating. Finally, the level of repeated fluoroscopy also significantly decreased after training. The data are shown in Table 1.

**Table 1:** Analysis of intraoperative communication level between surgeons and radiographers in pre‐training and post‐training

Group (rating out of 5)	Pre‐training	Post‐training	The difference [95% CI]	*p*‐value
Rating of intraoperative communication	3.64	4.037	0.398 [0.072, 0.725]	0.017*
Difficulty communicating with C‐arm mobile terminology	2.34	1.765	−0.572 [−0.880, −0.26]	0.001*
Difficulty communicating with the degree of C‐arm motion correction	2.52	1.926	−0.592 [−0.936, −0.24]	0.001*
Repeated fluoroscopy rate	2.84	2.06	−0.782 [−1.158, −0.40]	0.001*

### Fluoroscopy Times and Effective Rate

The four steps of the surgical technique are dissected, including placement, puncture, catheter drainage, and bone cement implantation. After surgeons and radiographers received terminology training, significant and lasting reductions in fluoroscopy time were observed. The most notable improvement was the shorter amount of time spent under fluoroscopy for the two surgical operations of puncture and bone cement implantation. Total fluoroscopy times decreased from 30.01 to 19.96 when comparing pre‐and post‐training indications.

Efficiency in fluorescence microscopy diagnosis. This study's training is helpful for increasing the precision of anteroposterior and lateral fluoroscopy since it greatly reduced the effective anteroposterior fluoroscopy rate and the effective lateral fluoroscopy rate afterward. By comparing the indicators of pre‐training with post‐training, the effective anteroposterior fluoroscopy rate increased from 46.5% to 75.7% (*p* < 0.001); the effective lateral fluoroscopy rate increased from 59.8% to 76.9% (*p* < 0.01). Therefore, this study can reduce the overall fluoroscopy times and improve the effective relative fluoroscopy rate (Table 2).

**Table 2:** Intraoperative fluoroscopy times in pre‐training and post‐training

Group (time in minutes)	Pre‐training	Post‐training	T‐value	*p*‐value
Positioning fluoroscopy time	4.79	2.21	2.341	<0.001*
Puncture fluoroscopy time	7.99	5.12	3.321	<0.001*
Fluoroscopy time of tube drainage	4.89	3.29	1.235	<0.001*
Fluoroscopy time of bone cement injection	12.34	9.34	0.175	<0.001*
Anteroposterior fluoroscopy time	11.24	9.25	2.986	<0.001*
Effective anteroposterior fluoroscopy time	5.23	7.01	2.472	<0.001*
Effective anteroposterior fluoroscopy %	46.5	75.5	1.3	<0.001*
Lateral fluoroscopy time	18.77	10.71	2.685	<0.001*
Effective lateral fluoroscopy time	11.23	7.24	0.243	<0.001*
Effective lateral fluoroscopy rate %	59.3	67.6	0.3	<0.001*
Total fluoroscopy time	30.1	19.96	3.758	<0.001*

### Transformation Time

After training, there was a notable reduction in the amount of time required to switch between the anteroposterior and lateral postures.

The results reveal that both the anteroposterior and lateral position transformation times reduced significantly after training, with the anteroposterior and lateral position transformation times decreasing the greatest, from 9.13 min before training to 5.24 min after training (Table 3).

**Table 3:** Intraoperative fluoroscopy time in pre‐training and post‐training

Group	Pre‐training	Post‐training	T‐value	*p*‐value
Anteroposterior fluoroscopy time (min)	9.24	7.01	2.971	<0.001*
Lateral fluoroscopy time (min)	19.23	15.21	0.283	<0.001*
Transformation time between anteroposterior fluoroscopy and lateral fluoroscopy (min)	9.13	5.24	0.765	<0.001*
Total fluoroscopy time (min)	37.6	27.46	2.569	<0.0018

### Repeated Puncture Rate

The percentage of patients requiring repeat punctures fell after participating in terminology instruction. When the arthroscopic fluoroscopy position is correct, but the lateral fluoroscopy position is too high or too low, the repeat puncture rate describes the likelihood of re‐puncture following adjustment to the anteroposterior position. There was a statistically significant drop in the rate of repeated punctures from 10.23% to 4.67% after participants received terminology training. Repeated pricking at both high and low points was reduced but at the former in particular (Table 4).

**Table 4:** Intraoperative puncture rate in pre‐training and post‐training

Group	Pre‐training	Post‐training	T‐value	*p*‐value
Repeat puncture rate	10.23	4.76	2.678	<0.001*
Rate of upper end of puncture point location	6.24	2.33	3.916	<0.001*
Rate of lower end of puncture point location	3.99	2.34	2.519	<0.001*

## Discussion

In this study, we found that smooth intraoperative communication between professional spine surgeons and radiographers can significantly lower the communication barrier, reduce the fluoroscopy frequencies and time, the rate of repeated puncture, and increase the effective fluoroscopy rate, all of which are important in improving the fluoroscopy in PVP. Multiple and effective intraoperative fluoroscopies can avoid or reduce the important complications of the PVP, such as spinal cord injury and bone cement leakage.^1^, ^13^, ^14^, ^15^

### Enhancing Communication Efficiency through Specific Fluoroscopy Terminology Training

Due to the lack of specific communication terminologies between the surgeons and the fluoroscopy radiographers, the communication efficiency between the two is reduced, which eventually leads to an increase in the number of fluoroscopies and a prolonged fluoroscopy time.^16^ Some studies proposed that using specific fluoroscopy terminology can improve the surgeons' satisfaction and reduce the work pressure of the fluoroscopy radiographers.^17^ Another recent study showed that excessive intraoperative radiation exposure increases the incidence of lung cancer and colon cancer in workers in the operating room. The maximum ambient dose equivalent rates for all considered distances and heights were observed at 330° and 30°. For example, the ambient dose equivalent rates at 1‐m distance and height of 1 m at 330° and 30° were 587 and 570 μSv/h, respectively, while the minimum value at 180° angle was about 11 μSv/h.^18^, ^19^, ^20^ Therefore, it is necessary to define specific fluoroscopy terminologies of PVP to enhance the communication efficiency between the operator and the fluoroscopy radiographers. The results of this study further showed that the effective anteroposterior fluoroscopy rate increased from 46.5% to 75.7% (*p* < 0.001); the effective lateral fluoroscopy rate increased from 59.8% to 76.9% (*p* < 0.01) and total fluoroscopy times decreased from 30.01 to 19.96 when comparing pre‐and post‐training indications, which confirmed that the training in specific terminology for surgeons and radiographers could further improve the communication efficiency and eliminated communication barriers.

### Nomenclature and Relative Movements of C‐Arm in the Study

In this study, the different active joints of the C‐arm are named according to the English letter sequence of “a‐b‐c‐d‐e”; the 12 directions of the C‐arm are divided into six groups of relative movements, which include: “arm up and arm down.” The movement of the arm up means that the arm body is raised vertically through the raising button of the C‐arm, the position of the vehicle body remains unchanged, and the raising distance is measured in centimeters. The movement of “arm down” is the opposite of the movement of “arm up,” and the “arm up and arm down” operation is required for both anteroposterior and lateral fluoroscopy. The movement of “arm in, arms out” means that the arm body moves forward in the direction away from the vehicle body; this movement is called “arm in.” The projection centre of the imaging system should overlap with the spinous process during anteroposterior fluoroscopy. At present, most of the C‐arm in clinical work use the laser positioning function to assist in the movement of an “arm in an arm out.” The movement of “rock up and rock down” refers to the movement to the head end of the patient is called “rock up,” and the movement to the foot end of the patient is called “rock down.” During the process of movement from rock up to rock down, the walking direction of the C‐arm shall be parallel to the patient's trunk, the moving distance is measured in centimeters, and the operation is performed by adjusting joint E.

### Surgical Considerations and Terminology for C‐Arm Movements

In this study, during the process of the movement of “rock up and rock down,” the surgeons should not only show the current fractured vertebral body but also look for specific L5‐S1 segments or vertebral bodies with obvious characteristics (wedge‐shaped deformation or characteristic osteophytes). The surgeons should not only refer to rib fluoroscopy imaging as the “gold standard” because many patients with rib development vary. The movement of “base up and base down” refers to the movement which is only used for anteroposterior fluoroscopy. By adjusting joint B, the tube on the trunk's ventral end moves to the patient's head end. The imaging system on the dorsal end of the trunk moves to the foot end of the patient, which is called “base up”; otherwise, it is called “base down.” The moving distance is measured in angle. Due to the existence of physiological kyphosis of the thoracic vertebra and physiological lordosis of the lumbar vertebra, the standard anteroposterior fluoroscopy requires the projection direction of the arm body to be parallel to the upper or lower endplates of the vertebral body. This study indicates that the high rate of repeated puncture before receiving training in this study is related to the fact that intraoperative fluoroscopy has not effectively adjusted the angle of movement of “base up and base down.” After training, the surgeons and fluoroscopy technicians can effectively communicate the fluoroscopy angle of movement of “base up and base down” and finally reach the standard fluoroscopy angle. The movement of “roll up and roll down” is only used for frontal fluoroscopy. By adjusting joint A, the tube on the ventral end of the patient's trunk slides towards the vehicle body, and the imaging system on the back end of the patient's trunk slides away from the vehicle body, which is called “roll up.” The moving distance is measured in angle and is the most commonly used for the transformation between anteroposterior and lateral fluoroscopy positions. However, we suggest that pronation supination is mainly aimed at patients with scoliosis, vertebral rotation, or vertebral rotation caused by patients' posture. By adjusting the movement angle of “roll up and roll down,” the standard ortho position requirements of spinous process centring and bilateral pedicle symmetry can be achieved. The movement of “swing up and swing down” is only used for lateral fluoroscopy. By adjusting joint E and joint C and with the vehicle body as the centre of the dot, the tube at the far end of the vehicle body moves to the patient's head end, which is called “swing up”; otherwise, it is called “swing down.” It is also used for patients with scoliosis to reach the standard that the arm body is parallel to the upper and lower endplates so as to display the endplates and pedicle structures.

In this work, we argued that the fluoroscopy duration is continuously transformed in the intraoperative design of the anteroposterior and lateral locations, despite the expected positive correlation between the fluoroscopy time and the number of fluoroscopies. Thus, the fluoroscopy time during intraoperative planning for both the anterior and posterior views was used as a benchmark in this investigation. In order to achieve standard fluoroscopy and effective fluoroscopy during surgery, the specific fluoroscopy terminologies defined in this study must ensure that the spinous process of the target vertebral body is centred, that the bilateral pedicles are symmetrical and equal, and that the upper or lower endplate of the vertebral body is parallel to the projection direction of the C‐arm. The specific target vertebral body (wedge‐shaped vertebral body) or L5‐S1 segment is shown, and its upper or lower endplate is parallel to the projection direction of the C‐arm. Without uniform fluoroscopy standards and the use of standardized fluoroscopy terminology, surgeons and fluoroscopy radiographers in the clinical setting are prone to making numerous invalid fluoroscopy errors.

### Strengths and Limitations of this Study

The most important advantage of this study is the C‐Arm fluoroscopy for the first application to obtain real‐time images. The time lag that may result as a consequence of the time required for the image retrieval from the conventional radiograph film cassette can have significant clinical ramifications, wherein the foreign body moves further deeper along the aerodigestive tract or into deeper tissue planes as a result of attempts at recovery or patient manipulation in the meantime. Despite the versatility of the intraoperative CT scan, the definitive cost‐effective technique to locate such large‐scale objects still remains elusive, which is worthy of extensive promotion in orthopaedic surgery.

### Conclusion

This study shows that smooth intraoperative communication between professional spine surgeons and radiographers can significantly lower the communication barrier, reduce the fluoroscopy frequencies and time, the rate of repeated puncture, and increase the effective fluoroscopy rate, which suggests training could greatly increase the effective fluoroscopy rate and significantly decrease the time needed to switch from anteroposterior to lateral positioning.

## Author Contributions

All authors had full access to the data in the study and took responsibility for the integrity of the data and the accuracy of the data analysis. *Conceptualization*, H. Y.; *Methodology*, H. Y., Y. X., and H. G.; *Investigation, H. Y. and A. X*.; *Formal Analysis*, Y. W. and H. Y.; *Resources*, Y. W. and H. Y.; *Writing – Original Draft* Y. X., H. G., and H. Y.; *Writing – Review & Editing*, Y. X., H. G., Y. W., and H. Y.; *Visualization*, Y. X., H. G., and H. Y.; *Supervision*, H. Y.

## Ethics Statement

Ethical approval for this study was taken from our institution, and written consent was taken from all patients.

## Data Availability

Contact the corresponding author for information related to data.
